# Feasibility and acceptability of technology-assisted problem management plus (TA-PM+) in community settings in Pakistan: a pre-post mixed-methods study

**DOI:** 10.1038/s41598-026-49596-8

**Published:** 2026-04-30

**Authors:** Maham Saleem, Shamsa Zafar, Fahad Abbasi, Hermann Pohlabeln, Siham Sikander, Daniela C. Fuhr, Hajo Zeeb

**Affiliations:** 1 Department of Prevention and Evaluation, Leibniz Institute for Prevention Research and Epidemiology - BIPS GmbH, Achterstraße 30, 28359 Bremen, Germany; 2 Leibniz Science Campus Digital Public Health, Bremen, Germany; 3 University of Bremen, Health Sciences, Bremen, Germany; 4 Fazaia Medical College, Air University, Islamabad, Pakistan; 5 University of Liverpool, Liverpool, UK

**Keywords:** Problem management plus, Technology assisted, Digital intervention, Lay health workers, Common mental health, Low-and middle-income countries, Public health, Depression

## Abstract

Problem Management Plus (PM+) effectively reduces symptoms of depression, anxiety, and psychological distress, demonstrating efficacy and cost-effectiveness in over 23 randomized controlled trials. However, real-world implementation through lay health workers often encounters significant challenges, particularly concerning intervention fidelity and feasible delivery. To address these challenges, Technology-assisted Problem Management Plus (TA-PM+), a digital adaptation of PM+, was co-developed. This study aims to evaluate the feasibility and acceptability of delivering TA-PM+ through government-employed primary healthcare outreach lay health workers known as Lady Health Workers (LHWs), in Pakistan. A pre-post, non-randomized feasibility study was conducted in Islamabad, Pakistan, involving 12 LHWs delivering TA-PM+ face-to-face to 77 women experiencing psychological distress. TA-PM+ is digital intervention designed to support LHWs in delivering five sessions of PM+, featuring psychoeducational videos for patients, automated assessments, and a guiding interface to enhance participant engagement and experience while ensuring intervention fidelity. Feasibility was assessed through session completion rates, participant retention, and qualitative interviews exploring implementation barriers and facilitators. Fidelity was evaluated using the ENhancing Assessment of Common Therapeutic factors (ENACT) checklist and digital automated session logs. Psychological distress was measured using the Patient Health Questionnaire-9 (PHQ-9) and Generalized Anxiety Disorder-7 (GAD-7) at baseline and post-intervention. Data analysis included descriptive statistics, paired t-tests, and thematic analysis of qualitative data. TA-PM+ demonstrated high feasibility, with 92% of participants completing all five sessions. Fidelity was high, with a mean ENACT score of 48 out of 54. Over 60% of the sessions met the minimum threshold with a mean session length of 45 to 50 min. Psychological distress showed preliminary improvements, with reduction in PHQ-9 (mean reduction: 6.07, *p* < 0.0001) and GAD-7 (mean reduction: 5.25, *p* < 0.0001). Participants valued structured sessions and psychoeducational videos. Identified challenges included internet connectivity issues, difficulties aligning session times with participants’ household responsibilities, and cognitive fatigue among health workers. TA-PM+ intervention delivery by government-employed primary healthcare outreach staff was feasible, acceptable, and preliminary effective in reducing psychological distress among women in a community setting in Pakistan. The digital adaptation maintained high fidelity and efficiently reduced session durations, indicating its strong potential for scale-up within primary healthcare systems. Further research is necessary to examine long-term outcomes and cost-effectiveness.

**Supplementary Information:**

The online version contains supplementary material available at 10.1038/s41598-026-49596-8.

## Introduction

Recent evidence indicates that mental disorders are among the leading global causes of disease burden, significantly contributing to years lived with disability, especially in low-and-middle-income countries (LMICs)^1^. In Pakistan, community prevalence estimates indicate high levels of anxiety and depression (27%), exacerbated by an acute shortage of mental health specialists, stigma, underfunded healthcare systems, and high out-of-pocket costs, resulting in a substantial treatment gap (90%) where most affected individuals remain untreated^2–4^.

Problem Management Plus (PM+) is WHO’s flagship brief psychological intervention. It is a brief, transdiagnostic psychological intervention designed for common mental health conditions and delivered by trained lay health workers^5^. Across more than 20 randomized controlled trials globally, PM + has demonstrated consistent effectiveness in reducing symptoms of depression, anxiety, and functional impairment^6–11^. A recent systematic review by Schäfer et al. further quantified these effects, reporting standardized mean differences of approximately − 0.46 for depression, − 0.51 for anxiety, and − 0.36 for functional impairment, all within the range typically interpreted as moderate effects. This review also provided evidence endorsing PM+ scalability and cost-effectiveness in adversity-affected environments^12^. However, scaling up PM+ remains challenging because it is a structured, competency-based intervention delivered over five weekly 90-minute sessions. This contributes to high attrition in routine settings and requires substantial training, ongoing supervision, and additional workload from lay health workers within resource-constrained health systems^6–10^.

Scaling up psychological interventions can involve leveraging non-specialist providers and digital platforms, to enhance accessibility and efficiency^13^. The incorporation of digital technologies plays a pivotal role in the scale-up process by facilitating delivery of therapeutic interventions, supervision, and outcome monitoring^13^. Digitisation is a strategy being used to scale up evidence-based interventions for mental health for LMICs^14,15^. Recent systematic review from LMIC contexts have indicated that digital mental health interventions delivered by non-specialists effectively address treatment and quality gaps by extending reach and enhancing competencies^16^. While digital delivery presents a viable pathway for scaling psychological interventions, gendered disparities in mobile access and digital literacy pose significant implementation barriers in LMICs^17^. In Pakistan women experience substantially lower rates of mobile phone ownership and internet access compared to men, constrained by socio-cultural norms and limited autonomy over device use^18^. National efforts to digitise the LHW programme, including development of electronic family registers for LHWs in Punjab, provide an enabling system-level opportunity for embedding digital mental health tools within established workflows^19^. Yet digitisation does not inherently promote equity and requires deliberate adaptation to local contexts when deployed as a scale-up mechanism.

To address these constraints, Technology-Assisted Problem Management Plus (TA-PM+), a digital adaptation of WHO’s Problem Management Plus, was co-produced with lay health workers, community women, and health system stakeholders through a three-stage participatory process^20^. Designed as a digital aid to support face-to-face delivery by LHWs, TA-PM+ platform directly addresses implementation barriers including limited digital literacy, restricted internet access, and workload pressures faced by female providers^20^. A theoretically grounded justification for TA-PM + can be derived from the Technology Acceptance Model (TAM), which posits that digital tools become accepted when they improve work performance and are easy to use^21^. PM+ necessitates lay health providers to consistently deliver problem-solving, behavioral activation, stress-management techniques, and strategies for strengthening social support, yet these components can be challenging to recall and implement with fidelity in routine practice^6,9,10^. TA-PM+ addresses these barriers by providing real-time guidance, automated sequencing of therapeutic steps and embedded audio-visual content that reinforces each component of PM+^20^. These attributes immediately augment perceived usability, the primary factors influencing digital adoption according to the TAM and facilitate the precise implementation of PM+’s active processes^21^. Results from our co-production and usability study showed that TA-PM+ achieved high usability and acceptability among lay health workers and clients, indicating that digital augmentation can support the delivery of PM + and standardise the delivery of core therapeutic components^20^. This study aims to test the feasibility, acceptability, and preliminary effectiveness of TA-PM+ intervention delivered by government employed community heath workers in Pakistan.

## Methods

### Design

A pre-post, non-randomized feasibility study design was employed to investigate key implementation constructs such as acceptability, feasibility, fidelity, and preliminary clinical outcomes of TA-PM+. Consistent with Medical Research Council guidance^22,23^, the study was not powered to detect between-group differences, and outcomes are interpreted as preliminary signals rather than causal effects. We use the CONSORT extension for pilot and feasibility trials as recommended by Lancaster & Thabane (2019)^24^(see Supplementary file 1) to report our feasibility study.

### Settings

The study was conducted in a semi urban area of Islamabad Capital Territory (ICT), in a Union Council (UC) Tarlai, that has a population of approx. 100,000^25^. UC Tarlai is a predominantly low-income and lower-middle-class area, with a significant number of migrants from different parts of Pakistan. The crude birth rate is estimated to be around 40 births per 1000 population. Among the poorer sections of the population, several adversities are common, including malnutrition, irregular income, and low literacy rates^25^.

The study area is served by a public Rural Health Center (RHC) and government-employed Lady Health Worker (LHW) Program. The LHW Program is currently operationalised under a geographical catchment area model in which LHWs (who are female community health workers) are assigned to households within an hour’s walk of their residence (roughly estimating to 100–250 households per LHW). A LHW delivers services to women only and provide health education and preventive/promotive maternal and child health care through monthly home visits^26^. There are 34 LHWs in UC Tarlai, and their catchment (each catchment area is based on a population of 1200–1700).

### Participants

As this study aimed to evaluate feasibility and acceptability, a formal sample size calculation was not performed, in accordance with methodological guidelines for feasibility research^22,23^.

The study participants were 77 adult women in the UC of Tarlai. To be included, participants had to be residents of UC Tarlai aged ≥ 18 years (self-reported) with (i) a Score ≥ 3 on the General Health Questionnaire (GHQ-12), a 12 item questionnaire of general psychological distress with a 4-point scale ranging from 0 to 3 scored bi-modally (0-0-1-1) when used as a screener (possible range 0–12); (ii) a Score ≥ 16 on the WHO Disability Assessment Schedule (WHODAS 2.0), a screener for functional impairment with 12 items measured on a scale ranging from 1 to 5 (possible range 12–60); (iii) were registered with LHW of the UC and willing to provide relevant data and have provided written informed consent. These screening thresholds (GHQ-12 ≥ 3 and WHODAS ≥ 16) are consistent with recruitment procedures used in previous PM+ trials in Pakistan, and all screening instruments have been translated and validated for use in the Pakistani context^6,7,27^.

### Recruitment

12 LHWs were randomly selected from the UC Tarali to deliver the intervention. Participants were adult women experiencing psychological distress, recruited from households registered with local LHWs. To minimise selection bias, approximately 20 households were randomly selected from the register of each LHW by the research team. LHWs approached potential participants based on randomly selected households from their catchment areas, inviting them to participate in the study. Women who agreed to be screened visited the LHW Health House on scheduled day, where all eligibility assessments and informed consent procedures were carried out independently by the research team. Screening continued until six eligible participants per LHW were enrolled.

### Intervention

TA-PM+ is a digital adaptation of the PM+ intervention, designed to enhance the fidelity and quality of delivery by LHWs through technological support^20^. It is a five-session psychological intervention designed to address common mental health conditions such as depression, anxiety, and stress. The details of its development are explained in a separate paper^20^. Each session is delivered weekly, face to face by LHWs using the TA-PM+ app and includes components like (i) stress management, (ii) problem-solving, (iii) behavioural activation, (iv) strengthening social support, and (v) relapse prevention. The digital platform supports these sessions with psychoeducational videos for patients, automated assessments, and guiding interface for LHWs. TA-PM+ enhances the engagement and experience of participants while allowing for the intervention to be delivered with fidelity.

### Procedure

Twelve LHWs participated in a five-day training program, which included instruction on the PM+ Urdu manual and additional five hours hands-on practice using the TA-PM+ digital application. Each LHW delivered five weekly face-to-face intervention sessions to participants, with each session lasting approximately 45–60 min. Intervention fidelity and delivery time-stamps were monitored through the TA-PM+ digital backend system by supervisors, who conducted weekly supervision meetings, initially in-person and subsequently online. Real-time technical support was available via a dedicated WhatsApp group to address any issues encountered during intervention delivery.

### Data collection

Quantitative data were collected at multiple time points: at baseline (pre-intervention), immediately following intervention completion after six weeks (post-intervention), and at five intervals during the intervention sessions. Demographic characteristics, including participants’ age, education level, and household income, were assessed at baseline. Additionally, qualitative data were collected post-intervention. Mental health and implementation outcomes were evaluated.

### Mental health outcomes

Depression symptoms were assessed pre- and post-intervention using the Patient Health Questionnaire-9 (PHQ-9), while anxiety symptoms were measured with the Generalized Anxiety Disorder-7 (GAD-7). Participants also self-reported their well-being before each session using the Psychological Outcome Profiles (PSYCHLOPS) tool. All tools have been translated and validated for use in Pakistan^28,29^.

### Implementation outcomes

#### Fidelity

Intervention fidelity was assessed using the ENACT checklist, a standardized tool with 18 items to assess the competence and quality of psychosocial intervention delivery by lay providers, each rated on a 3-point scale (0–2), resulting in a maximum possible score of 54. The checklist was applied to approximately 20% of the sample, with sessions randomly selected for each LHW, consistent with fidelity sampling approaches used in feasibility studies^30^. Additionally, quantitative data on session duration and completion rates and drop out rates were collected to further access fidelity.

#### Feasibility and acceptability

The feasibility and acceptability of the intervention were evaluated qualitatively using in-depth interviews and a focus group discussion with 28 purposively selected participants (12 LHWs and 16 intervention recipients). Maximum-variation purposive sampling was employed to ensure diversity in educational levels, socioeconomic status, levels of psychological distress, and variability in internet bandwidth available in their households, as internet signals influenced LHWs’ capacity to use the digital tool in the community. Sampling continued until information saturation was reached, defined as the point at which no new substantial insights emerged across the dataset, including within key variations in participant characteristics. Interviews were conducted face-to-face in privacy in Urdu by a trained female researcher at LHW health houses and participants’ homes, using a semi-structured interview guide (see Supplementary file 2) to explore participant experiences and contextual factors influencing intervention acceptability and feasibility. The interviewer was affiliated with the broader TA-PM+ development team but had no supervisory or evaluative role in intervention delivery. To minimise social desirability bias, neither participants nor LHWs were informed of her developmental role, and all interviews were conducted in private settings with emphasis on confidentiality, voluntariness, and independence from service delivery. Reflexive field notes were maintained throughout data collection.

#### Data analysis

Quantitative analyses were conducted using SAS (Cary, N.C.), while qualitative analyses were performed using MaxQDA. Descriptive statistics, including means, standard deviations, and percentages, were utilized to summarize participant characteristics and study outcomes. Given the exploratory nature of this feasibility pilot study, formal power calculations and sample size determinations were not conducted.

Paired t-tests were conducted to evaluate pre- and post-intervention changes in PHQ-9 and GAD-7 scores for those who completed baseline and follow-up assessment. All statistical tests were two-tailed, with an alpha level set at 0.05. The distribution of difference scores (pre-post) for both GAD-7 and PHQ-9 was visually assessed and found to approximate normality, justifying the use of paired t-tests for pre- and post-intervention comparisons. In addition, Wilcoxon signed-rank tests were conducted as a non-parametric robustness check to account for potential deviations from normality. To also account for the influence of covariates on the changes in both scores, we additionally analyzed the data with a linear mixed-effects model (LMM) using SAS PROC MIXED. The model included time point as a fixed effect, along with age, education, number of children, income and session duration as covariates.

The qualitative data were analysed using reflexive thematic analysis following the six-phase approach outlined by Braun and Clarke, which included familiarisation, generating initial codes, constructing themes, reviewing themes, defining and naming themes, and producing the report^31^. Two trained qualitative researchers conducted the coding, and both had been involved in the broader development of TA-PM+. Neither researcher held a supervisory role in intervention delivery, and their developmental affiliation was not known to participants or LHWs. To mitigate potential interpretive bias related to positionality, both coders maintained reflexive notes, met regularly to resolve discrepancies, and ensured that coding was grounded in the data rather than prior involvement. These procedures are consistent with best practice recommendations for managing positionality in qualitative health research^32^. The quantitative and qualitative findings were integrated through data triangulation.

## Results

### Study procedure

**Fig. 1:**
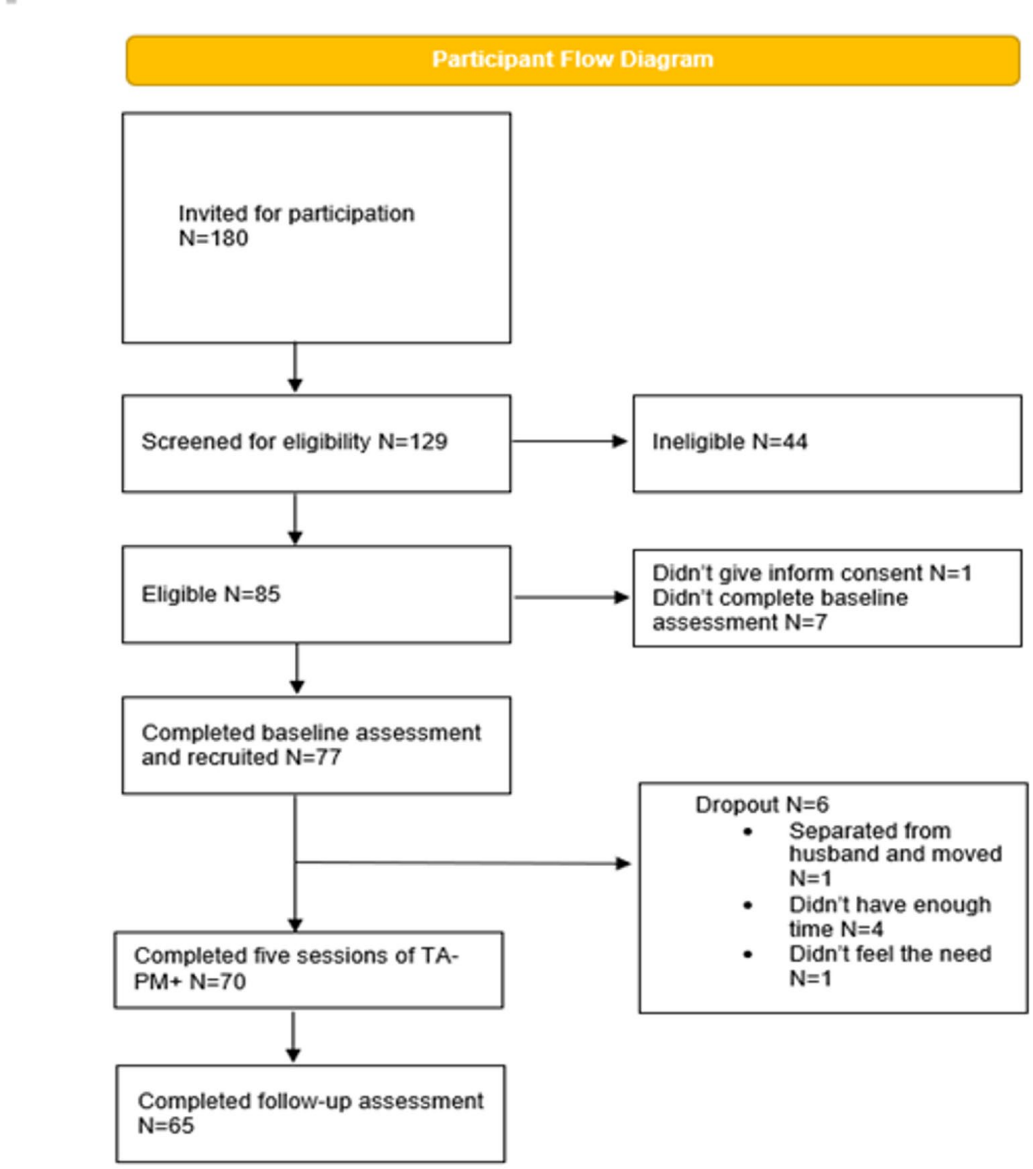
Participant flow diagram illustrating participant progression through the study.

Figure 1 illustrates the recruitment and retention flow of participants in the study. Of the 180 individuals initially contacted 129 agreed to take part in study and were screened. 84 were deemed eligible for participation. Of these, 77 consented and completed the baseline assessment. 71 participants completed the five sessions of TA-PM+ while 65 participants completed the follow-up assessments.

### Demographics

Table 1 presents the demographics. The study included 77 women participants with a mean age of 35.7 years (SD = 7.6). Educational levels varied, with 35.1% having 5–8 years of education, and household income levels showed 35.1% earning 20,000–25,000 PKR (about 70–90 USD) monthly. The intervention was delivered by 12 Lady Health Workers (LHWs), with average work experience of 16.4 years (SD = 3.2).

**Table 1:** Demographic characteristics of participants and lady health workers (LHWs).

Characteristic	Details
Participants (*n* = 77)	
Age	Mean: 35.7 years (SD: 7.6), Range: 21–55
Marital status	
Married	97.4% (*N* = 75)
Widow	2.6% (*N* = 2)
Number of children	
No children	9.1% (*N* = 7)
1–2 children	19.5% (*N* = 15)
3–4 children	50.6% (*N* = 39)
5 + children	20.8% (*N* = 16)
Education level	
< 5 years	18.2% (*N* = 14)
5–8 years	35.1% (*N* = 27)
9 years	28.6% (*N* = 22)
10 + years	18.2% (*N* = 14)
**Income**	
Below 20,000	15.6% (*N* = 12)
20,000–25,000	35.1% (*N* = 27)
25,000–50,000	29.9% (*N* = 23)
Missing	19.5% (*N* = 15)
Lady health workers (LHWs) (*n* = 12)	
Age	Mean: 48.0 years (SD: 3.6), Range: 41–53
Work Experience	
Years of Experience	Mean: 16.4 years (SD: 3.2)

Notes: N = Number of participants, SD = Standard Deviation, Income values are reported in Pakistani Rupees (PKR), Missing data indicates participants who did not provide income information.

### Implementation outcomes

#### Fidelity of TA-PM+

**Fig. 2:**
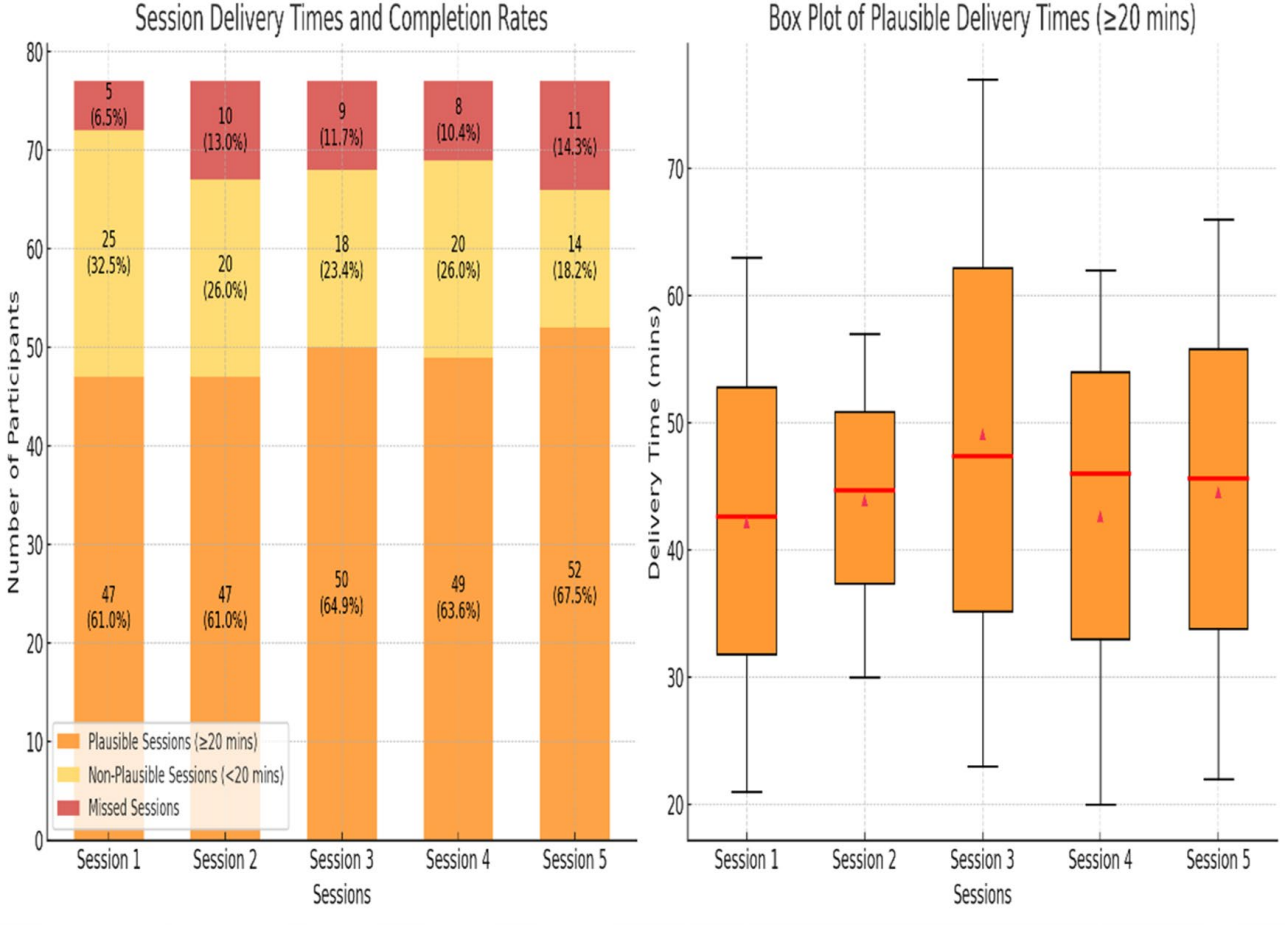
Session Delivery Times and Completion Rates. Left: Plausible (≥ 20 min), non-plausible (< 20 min), and missed sessions across five sessions. Right: Box plot of delivery times for plausible sessions (≥ 20 min). Box plots indicate medians and interquartile ranges; diamonds represent means with 95% confidence intervals.

The ENACT checklist scores (see Supplementary file 3) based on 20% of the sample, had a mean of M = 48 (SD = 0.6) out of a possible score of 54, indicating high adherence to the intervention protocol. Figure 2 presents the session delivery time, percentage of plausible and non-plausible delivery time and dropout in every session. Plausible delivery time in this study was defined as the minimum duration required to ensure all active components of the session were delivered. Sessions lasting less than 20 min were classified as non-plausible, meaning they did not meet the criteria to be considered a delivered session. Over 60% of sessions adhered to the plausible delivery time frame (≥ 20 min), increasing from 61.0% in Session 1 to 67.5% in Session 5. The median delivery time for plausible sessions consistently ranged between 45 and 50 min, reflecting adherence to the protocol. Delivery times showed considerable variability within sessions, as indicated by the spread of values around the median and the wide interquartile ranges in Fig. 2. Few sessions were missed, ranging from 6.5% in Session 1 to 14.3% in Session 5.

#### Feasibility and acceptability of TA-PM+

The feasibility and acceptability outcomes are reported below synthesized into key themes, their implications, and divided into LHWs’ and participants’ perceptions. A detailed thematic analysis, is provided (see Supplementary file 4). Feasibility and acceptability are presented as overarching themes were developed through a structured and interpretive analytic process. Feasibility reflected multiple analytic dimensions, including how TA-PM+ aligned with LHWs’ existing workloads, how easily the digital guide could be integrated into routine duties, and how contextual and household factors shaped session delivery for example, unpredictable availability of women, competing domestic responsibilities, childcare-related disruptions, restricted permission to visit health houses, and limited privacy for session engagement. Acceptability was informed by interpretive dimensions such as perceived usefulness and clarity of the digital guide, cultural and relational fit, comfort with the structured session flow, the rapport between LHWs and participants, perceived benefit of the intervention, variations in digital literacy, and access. These dimensions were derived from a detailed coding framework, presented in full in Supplementary File 4, which includes the themes, subthemes, and underlying codes used in the analysis.

### Lady health workers (LHWs)

#### Feasibility

LHWs found the intervention feasible overall, as the TA-PM+ app provided structured guidance for session delivery, helping maintain conversation flow and ensuring thorough topic coverage. The app’s structure and use of psychoeducational videos for patients were particularly appreciated for enhancing engagement and comprehension.

*“When we used to conduct sessions*,* we would sometimes forget certain things*,* but the videos explained everything in great detail*,* step by step. This made it easier for us to communicate because whatever we forgot was clearly explained in the videos. So*,* having the videos with us was a great benefit.” (FGD*,* LHW participant_6)*.

However, internet connectivity issues posed a significant barrier, particularly during session submission, which many found time-consuming and cognitively demanding. Scheduling sessions within the planned session timelines was another challenge due to patients’ unavailability. LHWs highlighted the frustration caused by repetitive questions of PSYCHLOPS in every session.

*“The same questions in every session became annoying*,* and patients often lost interest.” (FGD*,* LHW participant_1)*.

Despite these barriers, LHWs appreciated the support provided by online supervision, which offered practical solutions, such as submitting session timeslot screenshots during connectivity issues to the supervisors.

#### Acceptability

LHWs perceived the intervention as well-received in their communities, with videos praised for making the content relatable and actionable. However, LHWs also noted challenges in engaging patients from diverse socioeconomic backgrounds. While patients with manageable stressors showed interest, those with severe financial constraints or those who lived in abusive relationships were less responsive. Additionally, the absence of tangible benefits, such as monetary incentives or medical aid, was identified as a barrier to maintaining patient motivation.

Some LHWs expressed fatigue with the session workload along with their routine duties.

*“While delivering this intervention we also experienced what mental stress is " (FGD*,* LHW participant_2)*.

Nevertheless, LHWs believed their empathetic relationships with patients were a significant facilitator for the intervention’s acceptability.

*“They had closed themselves off*,* as if they had given up on life*,* but they have come out of that now. Through guidance by TA-PM+*,* we talked to them in detail*,* we realized that they still have something within them.” (FGD*,* LHW participant_10)*.

### Patients

#### Feasibility

Patients appreciated the structured sessions but highlighted challenges such as internet connectivity and household distractions. Sessions held at LHW health houses were better in terms of internet availability and privacy but often required patients to seek permission or adjust their schedules.

*“There were no internet issues at the LHW’s house*,* and the environment was peaceful compared to my home*,* where interruptions were frequent.” (Participant_12)*.

While some preferred 40–60 min sessions, others struggled with maintaining attention during longer sessions due to household responsibilities and lack of privacy affected session continuity, as many sessions conducted at patients’ homes experienced interruptions. A lot of participants reported that a more flexibility in terms of scheduling of appointment without the pressure of doing one session within one week. They expressed a preference for being able to complete sessions at their own pace, whether weekly, biweekly or even two sessions within single week. Despite these challenges, most patients found the sessions feasible to attend and reported that they can attend a 40–60 min session per week.

*“What I liked most was having someone to share my thoughts with. It felt like a weight was lifted from my heart and one can take out half an hour to three quarter of an hour for oneself.” (Participant_14)*.

Even though most participant deemed the session delivery format as feasible, some participants expressed the need to access videos later, citing household distractions that reduced active listening and a preference to watch the videos again when they are free.

*“Like how videos come to us on WhatsApp*,* for example*,* then we can watch them at any time. While watching the videos during session*,* my little kid was attracted and kept coming to me*,* so I couldn’t concentrate.” (Participant_15)*.

#### Acceptability

Most patients valued the intervention, particularly its focus on stress management and practical solutions. Many noted improvements in managing stress, dividing chores, and socializing better. Many found the strategies, particularly breathing exercises, effective in managing anxiety and improving daily functioning. Breathing exercises were the most practiced and most recalled strategy among patients. The psychoeducational videos and the opportunity to share their thoughts were especially appreciated.

*“When a person sees that someone else had a similar problem and solved it in this way*,* they feel encouraged to try it themselves*,* thinking*,* ‘I am just like them*,* so I can do it too. In the video*,* the results are immediately visible*,* so a person thinks*,* ‘If I do the same*,* I will also get better like that.” (Particiapant_2)*.

The intervention was also perceived as relatable, with its focus on practical solutions for domestic stressors. However, engagement was lower among patients facing severe socio-economic hardships, who felt their issues required broader systemic solutions. Women facing acute financial strain, heavy domestic workload or restricted autonomy described limited capacity to engage consistently, as immediate household and economic pressures took precedence over session attendance and intervention activities. This was also reported by LHWs that the absence of tangible support such as financial or medical assistance sometimes reduced participant motivation to continue.

*“To be honest*,* I initially thought*,* ‘Are we just wasting our time? What will we gain from this? My problems can’t be solved just by looking at a mobile*,* can they*,* therefore I didn’t feel like attending sessions.” (Participant_3).*

Despite this, most patients reported positive behavioural changes and advocated for the intervention to be extended to other community members.

#### Mental health outcomes

Mental health outcomes are reported in Table 2. Preliminary improvements were observed across all mental health measures. PSYCHLOPS scores decreased progressively across sessions, with mean scores dropping from 11.0 (Session 1) to 7.9 (Session 5), indicating reduced distress over time. For GAD-7, mean scores significantly decreased from 8.71 to 3.44, corresponding to a mean reduction of 5.25; SD 5.99 (*p* < 0.0001) suggesting improvements anxiety symptoms. Similarly, PHQ-9 scores dropped from 10.62 to 4.46, reflecting a mean reduction of 6.07; SD 7.73 (*p* < 0.0001). Standard deviations for GAD-7 and PHQ-9 change scores were relatively large, indicating variability in symptom change across participants. Despite this variability, the pre–post difference scores met normality assumptions. To account for potential deviations from normality, Wilcoxon signed-rank tests were additionally performed. Wilcoxon signed-rank tests, also showed significant reductions in both GAD-7 (S = 854, *p* < 0.0001) and PHQ-9 (S = 761, *p* < 0.0001), further supporting these indicative trends and aligning with the paired t-test findings.

**Table 2:** Changes in PSYCHLOPS, GAD-7 (Anxiety), and PHQ-9 (depression) scores.

PSYCHLOPS							
Session	*N*	missing	Mean	Median	Std Dev	Minimum	Maximum
Session 1: Dealing with Stress	71	6	11	11	3.7	1	20
Session 2: Dealing with Problems	70	7	11.1	11	3.2	5	19
Session 3: Get Going, Keep Doing	71	6	10.5	10	2.8	1	17
Session 4: Social Relationships	71	6	9.5	9	3.2	0	17
Session 5: Staying Healthy	70	7	7.9	8	3.2	2	14
**GAD-7 for Anxiety (Paired t-tests)**							
**Measure**	**N**	**missing**	**Mean**	**Std Dev**	**p-value**	**Minimum**	**Maximum**
Baseline (T0)	76	1	8.71	3.73	-	2	21
Follow-Up (T1)	68	9	3.44	3.47	-	0	12
Reduction (T0 - T1)	67	10	5.25	5.99	< 0.0001	−9	18
**PHQ-9 for Depression (Paired t-tests)**							
**Measure**	**N**	**missing**	**Mean**	**Std Dev**	**p-value**	**Minimum**	**Maximum**
Baseline (T0)	76	1	10.62	4.63	-	1	21
Follow-Up (T1)	68	9	4.46	4.73	-	0	20
Reduction (T0 - T1)	67	10	6.07	7.73	< 0.0001	−16	21
**GAD-7 for Anxiety (Wilcoxon Signed-Rank Tests)**							
**Measure**	**N**	**Mean**	**Std Dev**	**Signed-Rank Statistic (S)**		**P-value**	
Baseline (T0)	67	8.72	3.94	-		-	
Follow-Up (T1)	67	3.46	3.47	-		-	
Reduction (T0 - T1)	67	5.25	5.99	854		< 0.0001	
**PHQ-9 for Depression (Wilcoxon Signed-Rank Tests)**							
**Measure**	**N**	**Mean**	**Std Dev**	**Signed-Rank Statistic (S)**		**P-value**	
Baseline (T0)	67	10.60	4.80				
Follow-Up (T1)	67	4.52	4.73				
Reduction (T0 - T1)	67	6.07	7.73	761.5		< 0.0001	

Notes: PSYCHLOPS = Psychological Outcome Profiles, GAD-7 = Generalized Anxiety Disorder Scale (7-item scale, range 0–21; higher scores indicate greater anxiety), PHQ-9 = Patient Health Questionnaire (9-item scale, range 0–27; higher scores indicate greater depression severity), N = Number of participants, Std Dev = Standard deviation indicating variability of scores.

**Fig. 3:**
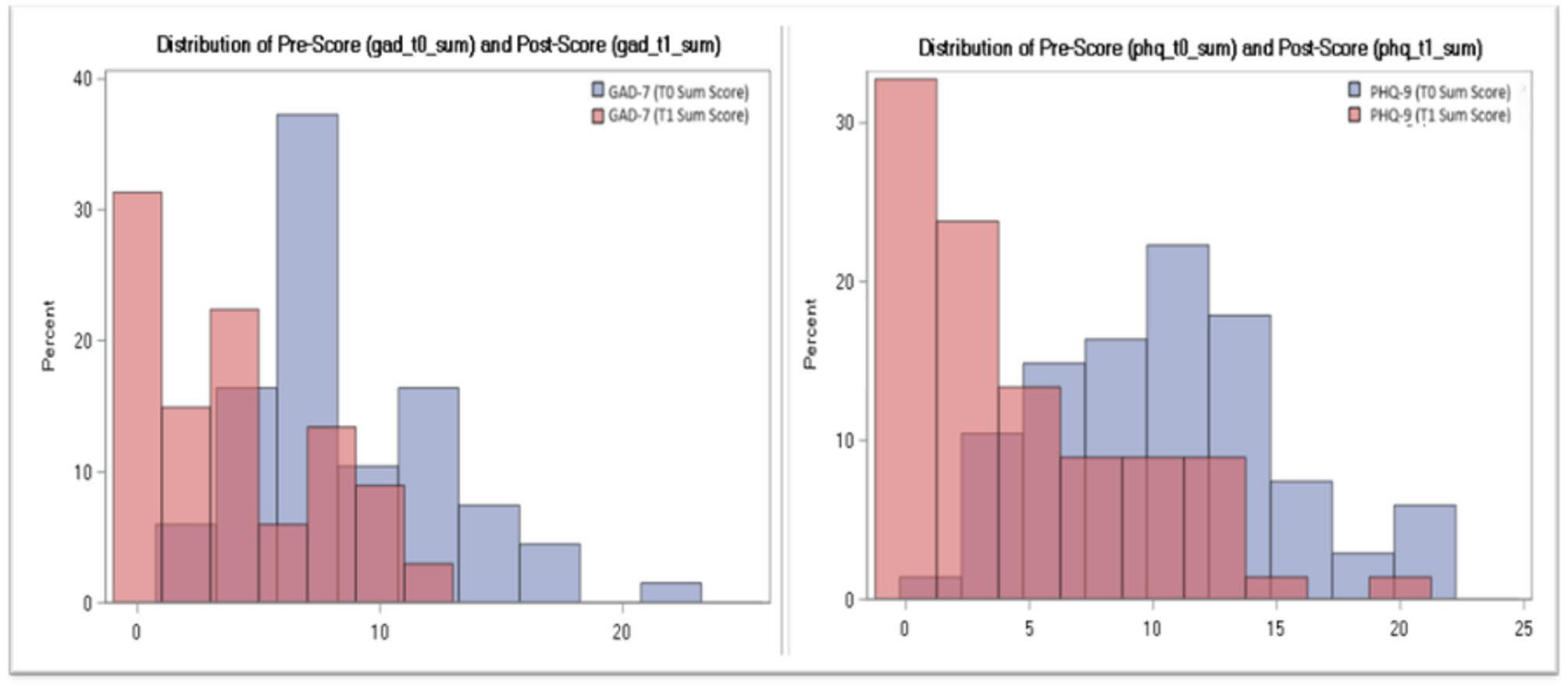
Distribution of pre- and post-intervention scores and score differences for GAD-7 and PHQ-12.

Figure 3 displays the histograms of the pre- and post-intervention score distributions for GAD-7 and PHQ-12, demonstrating a shift toward lower scores post-intervention.

#### Covariate analysis and sensitivity analysis

An additionally performed linear mixed model, accounting for covariates confirmed the significant reductions in scores from post- to pre-intervention, highlighting the intervention’s potential effectiveness (see Supplementary file 5). Notably, time point remained a significant fixed effect, while age, education, number of children, household income, and session duration did not significantly influence the outcomes, suggesting that symptom changes were consistent across participant characteristics and session lengths.

Two LHWs had missing digital session time-stamp data due to intermittent internet connectivity. However, all sessions were completed and verified through PSYCHLOPS entries, backend activity logs, and manual reporting to the supervisor. Because outcome data were fully available for the participants of these LHWs and all sessions had been delivered, we retained them in the main analysis. To assess the robustness of the findings, we conducted an additional sensitivity analysis excluding them (N =  56). This analysis showed reductions in PHQ-9 and GAD-7 scores that were comparable in magnitude and significance to the main analysis (see Supplementary File 5), indicating that the missing timestamp information did not influence the observed outcome patterns.

#### Triangulation of data

Triangulation analyses revealed complementary and explanatory findings for mental health outcomes and implementation processes (see Supplementary file 6). Quantitatively, GAD-7 and PHQ-9 scores showed significant reductions, with GAD-7 improvements appearing more consistent across participants. Qualitative data helped to interpret these trends by indicating that breathing exercises were the most practiced and perceived-as-helpful component, and were the only strategy recalled by all participants, suggesting a plausible mechanism for the stronger and more consistent anxiety improvements.

Triangulation indicated that TA-PM+ supported high-fidelity delivery while also highlighting data limitations. Approximately 10% of digital session time-stamps were missing for all the LHWs, and two LHWs had no time-stamp records due to internet connectivity issues, although their sessions were completed and documented through PSYCHLOPS ratings and manual reporting to supervisors. Participants of these LHWs demonstrated reductions in PHQ-9 and GAD-7 scores (see Supplementary file 6) comparable to the full sample. Qualitative findings similarly identified connectivity challenges as a barrier to timely data submission and highlighted that offline videos and automated session guidance helped maintain delivery fidelity.

## Discussion

The TA-PM+ intervention, delivered by LHWs in Pakistan, demonstrated feasibility and acceptability, with indicative improvements in PHQ-9 (mean change: 6.07, SD: 7.73) and GAD-7 scores (mean change: 5.25, SD: 5.99). These preliminary mental health changes warrant confirmation in future controlled studies. Reducing session duration from the traditional 90 min to 45–60 min proved feasible for both participants and LHWs, maintaining intervention fidelity, reflected in the mean ENACT score (48/54) and low attrition rates (7%). These findings suggest the potential of TA-PM + to improve scalability and effectiveness in resource-constrained settings.

TA-PM+ is an upscaled version of PM+, developed to address its implementation challenges. PM+ implementation barriers include issues of quality control in training, supervision, and delivery, compounded by the complexity of managing systematic stress and problem-solving protocols^6,9,10^. The integration of a digital platform including a frontend app for LHWs, a backend monitoring system for supervisors, psychoeducational videos, structured guidance, automated monitoring, and WhatsApp-based supervision streamlined delivery, reduced cognitive load, and ensured quality control, highlighting the intervention’s potential for scalability in resource-constrained settings.

In this feasibility study, preliminary improvements associated with TA-PM+ were similar in magnitude to outcomes reported in larger trials of traditional face-to-face PM+ interventions in community settings. Our TA-PM+ (feasibility) study demonstrated a substantial reduction in PHQ-9 scores, from 10.62 (SD = 4.63) at baseline to 4.46 (SD = 4.73) post-intervention. In contrast, a trial conducted in a conflict-affected region in Pakistan observed a decrease in scores from 10.11 (SD = 4.13) to 5.74 (SD = 4.83)^27^, whereas a study conducted in a disaster-affected region in Nepal^8^ reported a reduction from 12.7 (SD = 4.9) to 9.5 (SD = 5.0)^25^. These comparisons are descriptive only, as our non-randomised pre–post design does not allow causal inference. In terms of retention, 89% of participants in our study completed four or more sessions, compared to 82% (completing three or more sessions) reported in the trial in Pakistan and 98% in Nepal^8,27^. It is important to highlight that both trials involved group-based PM+ sessions, whereas TA-PM + was delivered through individual sessions.

Our findings also resonate with broader digital mental health implementation evidence in LMICs. A recent review highlighted infrastructural constraints such as weak connectivity, limited devices and insufficient IT support as systemic barriers to scale-up^33^. Consistent with this, LHWs in our study experienced intermittent internet bandwidth problems that affected real-time data upload, although the availability of offline videos, automated session guidance and WhatsApp-based technical support helped sustain fidelity. The Berardi et al. Study^33^ also emphasised that digital tools can enhance therapeutic engagement by supporting communication and structuring clinician–patient interactions. Participants and LHWs in our study similarly perceived that video-based psychoeducation and the structured digital flow improved clarity, rapport and perceived quality of care.

Gendered norms influenced women’s ability to engage in or attend sessions. Limited privacy within homes and competing caregiving responsibilities further constrained participation. Some women required household permission or faced restricted mobility, particularly when asked to attend a session at the LHW health house for improved privacy and engagement. Some women facing severe financial strain or intimate partner conflict expressed scepticism about the utility of digital interventions, stating that “a mobile video cannot solve my problem.” This aligns with evidence that digital interventions may feel less relevant or overly “rigid” to individuals whose distress is driven by structural adversity^33^.

TA-PM+ effectively addressed a critical barrier frequently reported for psychological interventions in the literature: the lengthy sessions, which are burdensome for both patients and providers, particularly in health systems where they significantly increase workload^6,9–11^. In this study, TA-PM+ demonstrated comparable effectiveness to conventional PM+ while reducing session durations to 45–60 min from 90, a timeframe deemed feasible by both patients and LHWs delivering routine healthcare services. Covariate analysis suggested that medium session duration (30–45 min) or long session duration (46–60) was not associated with differences in outcomes, indicating that session fidelity may be more important than session length. While further research through full-scale implementation trial is needed, this finding could revolutionize mental health care in LMICs by standardizing delivery via digital tools, enhancing fidelity, and reducing session times for integration into routine primary care.

The blended delivery model of TA-PM+ significantly impacts training and supervision. The rollout of TA-PM+ necessitated more hands-on training (approximately 5 h beyond the standard 70–80 h of PM+ training recommended in WHO guidance) to acclimatise LHWs to the digital workflow and in-session prompts. During ongoing delivery, the structured digital guide and backend monitoring features reduced the intensity of supervision required. Similar observations have been reported in digital mental health interventions, where automated guidance and real-time monitoring help maintain fidelity while decreasing reliance on frequent in-person supervision^15,16^. The automated identification of adverse occurrences and fidelity issues such as abnormally brief sessions or extended time between two sessions enabled supervisors to respond directly, when necessary, rather than depending exclusively on supervision sessions. These features enabled supervision to transition from weekly in-person meetings to one online session every two weeks. Consistent with findings from other digitally supported psychosocial interventions, these results suggest that while blended models may initially increase training demands, they can ultimately streamline supervision and support scalability in resource-constrained settings^14^.

### Limitation

This feasibility study has several limitations that should be acknowledged. The study was conducted in a single semi-urban area of Pakistan with a relatively small sample size, which may limit the generalizability of the findings to other settings. Future studies should aim to include more diverse samples from multiple regions to enhance external validity. Second, the reliance on self-reported measures for mental health outcomes may have introduced reporting bias, particularly given the face-to-face nature of data collection. Although these tools were validated for the local context, triangulating self-reported data with objective measures, such as biometric stress indicators, could strengthen future research. In addition, although password protection, encrypted transfer, and restricted system access were used, digital interventions inherently carry data-privacy risks that should be examined further in future studies.

The TA-PM+ intervention primarily targeted individuals with mild to moderate psychological distress. Its applicability and effectiveness for individuals with severe mental health conditions or complex social and financial stressors remains unclear. However, PM+ is not designed as intervention for clinical psychiatric disease. This limitation nevertheless suggests the need for additional research to tailor interventions for populations experiencing severe adversities. The study population consisted exclusively of women, both as LHWs delivering the TA-PM + and the participants receiving it. While this reflects the structure of Pakistan’s community health workforce, it limits the generalizability of findings to male beneficiaries or mixed-gender delivery models. Delivering a psychological intervention through LHWs from the same communities raises additional ethical considerations, particularly for women with trauma histories, including issues of privacy, boundaries, and comfort in disclosing sensitive experiences. Participant engagement may have been shaped by existing relationships with their LHWs, potentially influencing attendance and participation beyond the intervention itself. Fidelity was assessed in approximately 20% of sessions for each LHW; while this approach aligns with fidelity sampling practices in feasibility studies, evaluating a larger proportion of sessions would provide greater robustness. Finally, as this was an early-phase feasibility study focused on assessing feasibility and acceptability, no control group was included. Without a control group, the observed symptom improvements may also partly reflect natural symptom fluctuation, regression to the mean, Hawthorne effects, or concurrent life changes, and should therefore be interpreted as preliminary.

## Conclusion

The TA-PM+ intervention demonstrated feasibility and acceptability, with preliminary improvements in mental health outcomes. While these findings are encouraging, further research is needed to determine how TA-PM+ can be optimally integrated and sustained within routine primary healthcare systems. By leveraging digital tools, the intervention maintained high fidelity while reducing session durations, addressing key implementation barriers associated with traditional PM+. These findings underscore the potential of technology-assisted psychological interventions to enhance scalability, improve accessibility, and support integration into primary healthcare systems in resource-constrained settings. Further large-scale studies are needed to evaluate its long-term impact and cost-effectiveness.

## Supplementary Information

Below is the link to the electronic supplementary material.

Supplementary Material 1

Supplementary Material 2

Supplementary Material 3

Supplementary Material 4

Supplementary Material 5

Supplementary Material 6

## Data Availability

All data generated or analysed during this study are included in this published article [and its supplementary information files]. All data was anonymized to ensure privacy, and no identifiable participant information is included in the manuscript or supplementary materials.

## Abbreviations

LHS: Lady health supervisor
LHW: Lady health workers
LMICs: Low- and middle-income countries
PM+: Problem management plus
TA-PM+: Technology assisted problem management plus
UC: Union council
