# Supplementary material for: Feasibility and acceptability of technology-assisted problem management plus (TA-PM+) in community settings in Pakistan: a pre-post mixed-methods study
Source: Sci Rep. 2026 Apr 30;16:14153. doi: 10.1038/s41598-026-49596-8 (PMC13139419; doi:10.1038/s41598-026-49596-8)
Supplement: Supplementary file 2 — Supplementary Material 2 [file 41598_2026_49596_MOESM2_ESM.pdf]

Supplementary File 2: Qualitative Interview guide

INTERVIEW GUIDES

<u>WellPak Participant Interview Guide</u>

1. Greet person. Introduce yourself and introduce WellPak.
2. Obtain informed consent (note if person agrees to be audio recorded or not).
3. Do an ice breaker introduction activity so that everyone can know each person's name
4. Introduce study:
   You have been getting sessions of WellPak programme by Lady Health worker. Today, we want to ask you some questions about how you found it. We want to hear from you, what was good about it, and also what we can change to make it better in the future. Remember, we are not only looking for positive answers. We really want to know what can be improved and which things did not go so well, and we need to hear this from you, because you are the experts! We are planning to do the WellPak programme with more people, but we want to make it as good and as helpful as possible first. Remember, you don't need to answer all questions, and if you feel unable to answer a question please say so and we will move on to the next one.
5. Explain recording and confidentiality
   In order to remember what we talk about, we will we will record the interview. We will then write a report of what was said in this group. Your identity will be kept confidential meaning that we will not use your name or other identifying information in any of the reports or summaries of our discussion today.

   . Document:
   a. (note) Date of interview
   b. (note) Location of interview
   c. (ask) Age interviewee
   d. (observe) Gender of interviewee
   e. (note) Interviewee type (i.e. intervention participant (adolescent or caregiver), non-completer (adolescent or caregiver), facilitator, supervisory role) If a non-completer, how many sessions?
   f. Participant ID's
   g. (note) Interviewer initials
6. Turn on recorder and inform the participant that you are turning on the recorder and that the audio recording will start now.

Topic	Question	Additional Questions	Probes / RA Notes
About PM+in general	So first I would like to learn from you about what is the PM+ program. Could you describe it for me in your point of view?	*What kind of things did you learn there?*	*(USE THE FOLLOWING PROBES FOR ALL TOPICS):*<br>- *Please tell me more on that?*<br>- *Why?*<br>- *Can you explain that more to me?*<br>- *What do you think?*<br>- *Take some time to think.*<br>- *Is there anything else you can tell me about that?*<br>- *Do you have any examples?)*
Overall impressions about the experience	*Now, I want you to tell me how was your experience with the PM+ program WellPak program?*	*Tell me about what you liked about your experience?*<br>*Tell me about what you did not like about your experience?*<br>*What was your experience with the LHWs?*<br>- *What did they do well?*	*Probe throughout on reasons for liking or disliking an aspect, and suggestions for improvement*

		- *What could they do better?*<br><br>*What was it like for you to be given an intervention using the digital Tablet?*<br><br>- *What did you like?*<br>- *What did you not like?*<br><br>*What do you think of the duration of the sessions and the programme in general?*<br>*Can you describe the content of the program (like the different methods that were part of the sessions)*	Probe on positive and negative views about the duration.<br><br>Probe on positive and negative views of the program.
	I'm interested to know more about the different activities in WellPak	*What specific activities did you find most helpful in the different sessions? [list at least 3 components]*<br>*What specific activities did you find not so helpful in the different sessions? [list at least 3 components]*	*Probe on the Breathing exercises, problem solving , behavioural activation for breaking inactivity cycle, strengthening social relationships)?[list at least 3 components]*
Video	*Okay, you've told me a lot about your experiences, but now I want to know a bit more about the videos that LHWs showed you for each session. So can you describe your experience of the WellPak videos (*	*Tell me about what you thought was good about the Videos that were developed for you?*<br>*Tell me about what you thought was not so good about the videos?*<br>*Was the duration and the information provided in the videos easy to understand?*<br>*What are your thoughts about the language used in the videos?*<br><br>*As we have mentioned, we want to make the program better and your opinion is very important to us, so if you wanted to improve the videos for other people here, what would you change?*	*[If NO NEED FOR IMPROVEMENT:] are you sure there is nothing that you would like to change about the videos? It would be very helpful for us to understand your thoughts so that we can improve the program for others.*<br>Probe on positive and negative views of the materials.
	Tell me about any parts of the programme that did not make sense, or that you thought were not relevant or that you do not think is acceptable to people in your community?	*Are there any parts of the WellPak programme that you think another people would have trouble understanding, would find irrelevant, or unacceptable?*	*[If NO:] are you sure there is nothing that you would like to change about the programme? It would be very helpful for us to understand your thoughts so that we can improve the program for others.*
Involvement of family/friends	*Describe any times that you provided your family or friends information about the WellPak?*	*What did you tell your family or friends about the WellPak programme?*<br>- *[If you told them], what were their impressions about the programme?*	

		In what other situations would you talk to other than your family or friends about the program?	
Helpfulness / impact	*Describe how you found implementing the skills the programme taught to you in your everyday routine?*	*How easy or difficult was it for you to use the skills the programme taught you in your everyday life? [example: breathing exercise, solving a problem, being active and doing little things in daily routine to break inactivity cycle, meeting people outside]*<br><br>*Tell me about anything that got in your way of using the skills that the program taught you? If so, what was it?*<br>*Were the videos helpful in understanding the strategy and skills that were taught to you?*<br>*Were there skills that you practiced alot?*<br>*Can you give me an example of how you included it in your routine?*	*Why or why not?*<br><br>Explore barriers and facilitators to skills development
	Compared to before you joined the group, have you noticed any changes in your ability to manage any distress you felt?	*Explain how the program helped you to manage any distress you felt? And in what way?*<br>- *Give me an example of how the programme helped you?*<br><br>*How are you dealing with your problem(s) compared to how you were dealing with them at the beginning of the program?*<br>*Do you think that the programme could help another women in Pakistan to manage their distress? And in what way?*	
	How, if at all, did taking part in the programme impact your wellbeing (how you feel about yourself)?	*What, if any, changes have you noticed in how you feel since joining the programme? (like: your mood, feeling good, feeling bad etc.)*	
Intervention adherence	*Now I want to know more about how it was for you getting to the sessions and using the skills.*<br><br>*Describe how easy or difficult you found getting weekly sessions?*<br><br>*Describe how you felt receiving the intervention*	- *Tell me about anything that made it difficult for you to attend? If so, what was it?*<br>- *Tell me about anything that made attending easy or encouraged you to attend? If so what was it?*<br>- *Tell me if watching and understanding the videos difficult for you? If so, what made it difficult*<br><br>*How do you feel about the LHWs using the digital app while delivering the intervention to you?*<br>*How could we make it easier for people to get all the sessions?*	Probe on barriers and facilitators to attendance.<br>If nothing was shared inquire about whether it was because of:<br>- The Location<br>- Work issues<br>- Taking care of kidss<br>- Helping caregivers at home<br>- Conflict with other schedule<br>- Too much time required<br>- Etc

	while LHWs used the digital Tab		[If they did not mention anything about digital app of LHWS please ask about it directly].
Overall suggestions	*We've talked about a lot already, but if you wanted to improve the program , are there any other things that you would change?*	*What might the other programme participants have thought could be improved about it?*	
Review the questions with the interviewees still present. If anything is not clear, ask for clarification			
Ask the interviewees if they have anything to add	*Is there anything that you would like to tell me that we have not already discussed?*		

WellPak LHWs Interview Guide

1. Greet person. Introduce yourself and introduce WellPak
2. Obtain informed consent (note if person agrees to be audio recorded or not).
3. Introduce study:
   You have recently participated in the WellPak programme as a facilitator. We would like to ask you some questions about your experience of this programme to help us to think about how it could be improved for delivery in the future. There are no right or wrong answers to the questions we are going to ask. We will be speaking to a number of people, asking everyone the same questions. If you feel unable to answer a question please say and we will move on to the next one.
4. FGD: Group Agreement: Put some rules to the session: if anyone wants to speak to raise their hands, to not cut each other while someone is talking especially that we are recording. No judgement, respect the privacy of the group, etc.
5. Document:
   a. (note) Date of interview
   b. (note) Location of interview
   c. (ask) Age interviewee
   d. (observe) Gender of interviewee
   e. (note) Interviewee type (i.e. intervention participant (, facilitator, supervisory role) If a non-completer, how many sessions?
   f. Participant ID’s
   g. (note) Interviewer initials
6. Turn on recorder and inform the participant that you are turning on the recorder and that the audio recording will start now.

Begin the FGD:

**Topic**	**Question**	**Additional Questions**	**Probes**
Overall impressions	*Describe your experience of delivering the WellPak PM+ intervention?*	*Tell me about what was positive about your experience overall?*<br><br>*Tell me about what was negative about your experience overall?*	***(USE THE FOLLOWING PROBES FOR ALL TOPICS: Can you expand a little more on that?***<br><br>***Is there anything else you can tell me about that?***

		If you wanted to improve the program for other LHWs, what would you change?<br><br>*If we were to bring PM+ to LHWs scope of work, what kinds of problems do you think we might encounter? How could we solve these problems?*<br><br>*What people of the community have thought could be improved about it?*	***Do you have any examples?)***
Overall experience with the digital App	*Describe your experience with the digital app of WellPak (TA-PM+)*	*Tell me about what was positive about your experience with the app?*<br><br>*Tell me about what was negative about your experience with the app?*<br><br>*If you wanted to improve the app for other LHWs, what would you change?*<br><br>*If we were to bring this app to LHWs scope of work, what kinds of problems do you think we might encounter? How could we solve these problems?*	
	Describe your experience of the WellPak materials (videos / manual)?	*Tell me about what you thought was good about the videos?*<br><br>*Tell me about how you think the videos could be improved?*<br><br>*If you wanted to improve the videos for other facilitators, what would you change?*<br><br>*Tell me about what you thought was good about the manuals?*<br><br>*Tell me about how you think the Manuals could be improved?*<br><br>*If you wanted to improve the manual for other LHWs, what would you change?*	
Facilitation	*Were there any difficulties you found about delivering intervention to the participant?*	*What did you think of the time to deliver the intervention to participants?*<br><br>*How helpful do you think was wellpak App to deliver the intervention*<br><br>*What did you find most challenging or burdensome about delivering the intervention through app?*<br><br>*What did you find most rewarding about delivering the intervention through the app?*<br><br>*What sorts of things would make delivering this easier?*	

		What kind of support would you suggest providing to facilitators given these difficult aspects of the job?	
Language	*What sorts of language issues came up while you were facilitating?*	*How did you handle these issues?*<br><br>*How can we improve the process of translation to avoid having these issues in the future?*	
Digital App and delivery of session	*What sorts of issues came up while you were delivering the intervention using the app?*	*How did you handle these issues?*<br><br>*How can we improve to avoid having these issues in the future?*	
Training and supervision?	*Describe your impressions of the training you received in becoming an WellPak facilitator?*	*How prepared did you feel for your role of facilitator at the end of training?*<br><br>*How can the training be improved?*<br><br>*Which aspects of the training did you think were most helpful?*<br><br>*If your colleague or friend were training to be a facilitator, how would you improve the training and supervision so that they would get the maximum benefit from this process?*	**Probe on both**<br>**PM+ training**<br>**TA-PM+ training**
Most useful components	*Which parts of the programme were most helpful for participants?*	*How were these parts helpful?*	**Probe on different aspects**<br>**Sessions**<br>**App**<br>**videos**
	Tell me about any parts of the programme that did not make sense, or that you thought were not relevant or acceptable to people in this community?	*How were these parts unhelpful or potentially harmful?*	**Probe on different aspects**<br><br>**Sessions**<br>**App**<br>**videos**
Review the questions with the interviewee still present. If anything is not clear ask for clarification			
Ask the interviewee if they have anything to add	*Is there anything that you would like to tell me that we have not already discussed?*		
