# Supplementary material for: Feasibility and acceptability of technology-assisted problem management plus (TA-PM+) in community settings in Pakistan: a pre-post mixed-methods study
Source: Sci Rep. 2026 Apr 30;16:14153. doi: 10.1038/s41598-026-49596-8 (PMC13139419; doi:10.1038/s41598-026-49596-8)
Supplement: Supplementary file 3 — Supplementary Material 3 [file 41598_2026_49596_MOESM3_ESM.pdf]

Supplementary file 3: ENACT checklist score

ENACT Score for Lady Health Workers (20% of sample)

Variable	N	Mean	Std Dev	Minimum	Median	Maximum
LHW age	12	48	3.6	41	49	53
Work experience	12	16.4	3.2	14	15	24
Duration	12	45.1	1.4	43	45	48
Enact score	12	47.8	0.6	46	48	48
