# Supplementary material for: Feasibility and acceptability of technology-assisted problem management plus (TA-PM+) in community settings in Pakistan: a pre-post mixed-methods study
Source: Sci Rep. 2026 Apr 30;16:14153. doi: 10.1038/s41598-026-49596-8 (PMC13139419; doi:10.1038/s41598-026-49596-8)
Supplement: Supplementary file 4 — Supplementary Material 4 [file 41598_2026_49596_MOESM4_ESM.pdf]

Supplementary File 4: Qualitative Analysis code chart

Theme	Description	Subtheme	Codes	Quotes
**PM+ perception of participants**	*This includes participants' perceptions of the PM+ intervention, focusing on their views about its relevance, and applicability in daily life. It also includes the specific strategies participants recalled from the intervention,*	**Participants perception about TA-PM+**	Managing domestic problems	This was about how we used to live with mental stress regarding children, household matters, or other issues. Lady Health Worker regularly visits our home and advises us that we should exercise, socialize, and integrate with others. (Participant 4)
				What the Lady Health Worker told us was that if we feel tired doing all the work at once, we should break it into smaller parts. This way, we won't get exhausted, and the work won't pile up either. (Participant 6)
			For womens Health, stress and depression	This is about health, such as exercise, and guide us about relaxation, teaching us how to relax our minds. (Participant 7)
				This is about someone experiencing mental stress due to domestic issues, which are common in every household. The Lady Health Worker has given us some sessions regarding this. (Participant 2)
		**Participant recall of TA-PM+ strategies taught by LHWs**	Divide workload in smaller parts	They said that if you get tired from working continuously, take breaks and do the work bit by bit so you don't get exhausted. This way, the workload won't pile up." (Participant 6)

			Breathing exercise to feel better	“…LHW would ask about what was causing pressure or stress on your mind, and then suggest exercises to help you relax.” (Participant 7)<br>“LHWs were explaining that for tension, you should hold your breath for some time — maybe for a minute or so — and then take deep breaths. This will give you relief.” (Participant 14)
			Share problems and feelings with others	The LHW said that you should share your worries with someone — with your husband, the people you spend time with, or your parents. Similarly, you can visit a neighbor, sit with them, and talk for a while. It will make you feel better." (Participant 13)
			Strengthen social relationship	"The LHW advised to go outside, participate in some activity, chat with friends, or arrange tea gatherings... this will help change your environment." (Participant 2)<br><br>"The LHW used to say, 'Don't lose hope. If you're feeling tense, go outside or have a conversation with someone. When your mind is at peace, you'll naturally start to see solutions to your problems.'" (Participant 10)
		**Most practiced strategy**	Breathing exercise	"When I sit down, I definitely do it." (Participant 10)<br>"Yes, I remember this (breathing exercise) because I practice it. Those who don't practice it don't remember." (Participant 12)<br>"Now, when the LHW leaves for her home, and we are lying down or sitting

				on the bed, we relax and do such exercises." (Participant 4)
**TA-PM+ Session's delivery**	*This includes details of the TA-PM+ sessions, specifically their duration, nature, and frequency, and place as recalled by participants and LHWs.*	**Session delivery Place reported by participants**	Conducted at home	No, the Lady Health Worker herself used to visit us. She guided us by saying, "Do it this way, do it that way." (Participant 12)
			Conducted in health house	No, they never gave TA-PM+ session at home. We came here (to health house), and the videos were also shown here. She visits homes just generally, as she is our Lady Health Worker. (Participant 5)
			Conducted at home and health house	I also keep visiting here (Health House), and she also keeps going to our house. (Participant 3)<br><br>"We used to come here (Health House) as well, but if we didn't come, the Lady Health Worker would visit us at home." (Participant 14)
		**Session delivery place reported by LHW**	Most sessions at Health House	We conducted it at the Health House. Yes, most people had their sessions at the Health House. (LHW Participant 7, Focus Group Discussion)
			Few sessions at patient place	I conducted the sessions at their homes. I only invited them to my house when your team visited; otherwise, all sessions took place at their homes. (LHW Participant 4, Focus Group Discussion)
		**Session delivery format as reported by Participants**	Question and answer about health/mental health/routine life	They would ask us about our problems, what benefits we gained from the previous sessions, how we were feeling, what our daily routines were, if we were mentally well, if our minds

				were alright, or if we were experiencing any stress. We discussed these kinds of topics. (Participant 11)<br><br>They had certain questions that they would ask us, and we would answer based on our situation or daily life experiences. If everything was fine, that was good; otherwise, they would advise us on what to do. (Participant 14)
			Discussion about problems	These were the kinds of things we discussed, often about mental stress. We would share our tensions with the Lady Health Worker, telling her about the stress we felt during the month. (Participant 7)
			Videos and discussion	In that, we watched exercises and similar things in the videos, and the Lady Health Worker also taught us these exercises. First, she would ask if we had any problems or mental stress. (Participant 9)<br><br>In every session, she first listened to our stress or problems, then showed us videos, and afterward, she helped us apply those exercises practically. (Participant 7)
		**Session frequency and time as reported by patients**	Session time varied from 30 minutes to an hour	I'm telling you that sessions could last between half an hour to 45 minutes, sometimes even an hour, especially when she showed us videos and explained them herself. She also used to operate the app on her mobile phone. (Participant 2)

				was probably around 30 minutes; I never really noted the exact time. (Participant 9)<br><br>An hour would easily pass while talking, thinking, and understanding things. When something is for our benefit and improvement, an hour doesn't feel long at all. (Participant 10)
			Session was 15 to 20 minutes	They would sit for around 15 to 20 minutes, continuously doing this. (Participant 14)
			Weekly sessions	She used to visit our home once a week, continuously for four weeks. She came weekly and provided guidance each time. (Participant 4)
		**Session duration, frequency and numbers as reported by LHWs**	Five weekly sessions were conducted with each patient	here were five sessions each—five patients received five sessions, with each session conducted weekly. (LHW Participant 7, Focus Group Discussion)
			45 to 60 minutes long session	It usually took about 45 to 60 minutes. Sometimes we even spent a few extra minutes, but the time we spent on the app itself was shorter. (LHW Participant 9, Focus Group Discussion)
**Feasibility of TA-PM+**	*This includes participants' and LHWs' perspectives on the feasibility of TA-PM+ sessions, specifically the duration, nature, and frequency of the sessions conducted. It also includes their experiences regarding factors that acted as barriers or facilitators to session delivery.*	**Challenges for participants in Home-Based Session**	Disruptions Due to Childcare Responsibilities	Yes, the sessions were conducted quickly. You know how it is—when I sit with my mobile, one child comes from one side, and another from the other. The children keep running after the mobile. (Participant 3)
			Limited Privacy for Session Engagement	The sessions lasted about 15 to 20 minutes because I have children, and as you know, my in-laws live with us too, so I couldn’t get much privacy. (Participant 15)

			Challenges in Availability and Time Management for Participants	Yes, LHW keeps coming. Let me tell you, she keeps calling me, but I'm often not at home. Sometimes I'm here, sometimes I'm somewhere else. Even now, she called me to come because I was out somewhere, far away. (Participant 3)<br><br>Actually, LHW has the time and makes the effort to come for us, but for us, it's a bit difficult to manage. (Participant 3)<br><br>It was just that if, God forbid, the session went over 40–45 minutes, we would start thinking that it should end soon, and the Lady Health Worker should leave. (Participant 2)
		**Barriers to Participation in Health House-Based Sessions for participants**	Restricted Permission for Health House Visits	Yes, there was an issue with getting permission to attend the sessions. So, I would secretly come anyway, but still, it didn't feel right since they were visiting. I would make an excuse, like going out to buy vegetables, and then attend the session. (Participant 5)
			Time Constraints for Health House Attendance	The difficulty is that even now, the Lady Health Worker has already visited my home twice to call me, right? The thing is, we live in a joint family, and as you know, it becomes very difficult to step out or to give time to someone. (Participant 14)
			Stress from Neglecting Household Duties During Sessions	I am alone here, responsible for all the household duties. When there is stress from household work, I can't really

				focus on these things. It's the same thought—one wonders what the point siting in health house and just listening. (Participant 3)
		**Barriers to session delivery for LHWs**	Participants' Unpredictable Availability	It wasn't too difficult for us, but if our clients weren't at home, we had to make three or four visits. (LHW Participant 8, Focus Group Discussion)<br><br>When the women were not at home—sometimes they were out, had a sick child, or attended a wedding—our sessions would get delayed. A session meant for one week could extend to two weeks, sometimes even three. Once, a participant had a delivery in between as well. (LHW Participant 3, Focus Group Discussion)
			Difficulties in Scheduling Sessions with Women in Home-Based Settings	Sometimes, the women couldn't give us proper time. They had different issues at different times. The time we scheduled with them often didn't work out, and we couldn't find them when needed. We had to ask multiple times, repeatedly contacting them to check when they would be available and free. (LHW Participant 5, Focus Group Discussion)
			Delays in Participant Attendance for Health House-Based Sessions	We used our own Wi-Fi to connect and work. One major issue was that women couldn't reach our homes on time as scheduled—they would come with great difficulty. We had to wait for them and sometimes even go to their homes to call them. This was quite a challenge. (LHW Participant 2, Focus Group Discussion)

		**Technical Issues in using tool**	Problems in session submission due to weak or no internet	There were issues, possibly with the internet, where our sessions wouldn't submit." (LHW Participant 10, Focus Group Discussion)<br><br>"Our sessions wouldn't submit properly. Sometimes we had to submit a single session two or three times. There were many such issues." (LHW Participant 5, Focus Group Discussion)<br><br>"When we visited participants' homes, there were signal issues. Although the session would appear to be submitted, when they later checked our records, nothing was there." (LHW Participant 3, Focus Group Discussion)<br><br>"The biggest issue was that the application wouldn't work properly due to poor mobile network signals inside people's homes." (LHW Participant 8, Focus Group Discussion)
			Questions were repetitive, increased session duration and irritates patients	"The same questions were asked in every session. There was only a slight change, but it was still mostly the same questions, which didn't feel good — like they were just repeating the same things." (LHW Participant 4, Focus Group Discussion)<br><br>"We would ask once, 'What issue did you face last week?' Then there would be another question, followed by three or four more steps like, 'Okay, apart from this, is there any other issue?' After that, it would continue, 'Alright, any other problem besides this?' The

				person would eventually respond with frustration, saying, 'What is this? I've already told you once, and now they're just going on and on...'" (LHW Participant 5, Focus Group Discussion)
**Acceptability of TA-PM+**	*Participants' and LHWs' acceptability of TA-PM+ includes participants' overall experiences with the digital tool, usability, ease of interaction, and engagement with the technology. Participants' experiences with LHWs, including quality of interactions, support, guidance, and rapport-building during the intervention, are described. Parts of the intervention identified as unacceptable or problematic by participants.*<br>*LHWs' experiences with the TA-PM+ app and supervisors, supervision quality, support received, and training adequacy are included. Parts of the intervention identified as unacceptable or problematic by LHWs.*<br>*The perceived impact of TA-PM+, particularly improvements in participants' emotional well-being, stress management, coping strategies, and daily functioning, is also presented.*	**Participants Experience with digital tool**	TA-PM+ enabled follow up assessment by LHWs	From that, the LHW would ask, 'Have you felt any difference from the advice given to you? Did you try any of the suggested activities? If not, why? Did you notice any change…?'" (Participant 2)
			Vidoes demonstrated breathing exercises	The LHW would say, 'Do this exercise like this,' and they would play a video... The video showed how to do it, step by step — sitting with others, doing this and that — they were teaching a lot of things." (Participant 3)<br>the doctor was explaining the method — first place your hand on your navel, then take a deep breath... Then the LHW would guide us to repeat this three or four times continuously." (Participant 4)
			Videos taught stress coping strategies	I saw that video — it talked about household problems, physical issues like body aches, feeling fatigued, or moments when you feel mental distress. They showed videos explaining these things as well." (Participant 3)<br>Videos was about depression and such issues — about patients who experience depression." (Participant 12)

		**Patient Experience with LHWs**	Empathetic listening	..They treated us very well. It was a very friendly environment, just like someone from your own family. Just like a mother holds her child's finger to teach them how to walk — exactly like that." (Participant 2)<br>listening to concerns, then responding with empathy. It wasn't about mocking or criticizing a woman by making judgmental remarks." (Participant 7)
			Problem solving approach of LHW	Recently, we had an issue at home involving my sister-in-law. We shared it with the LHW, who guided us well. She also explained things to my in-laws, and following her advice improved the situation at home." (Participant 15)
		**LHW's experience with tool**	TA-PM+ Driven Structured Conversation Flow	We would come to that topic while using the questionnaire and app." (LHW Participant 8, Focus Group Discussion)<br><br>"There was a video they had included for us named "session aid" for every session, kind of like a guide. If we forgot something, we could watch it, and it covered everything — just like how we were supposed to deliver the session. We would watch it and revise our content accordingly." (LHW Participant 2, Focus Group Discussion)<br><br>"Whenever we found something difficult, we would see the flow charts and different icons in a session take guidance from it." (LHW Participant 6, Focus Group Discussion)
			TA-PM+ Facilitated Identification of	"We received their genuine concerns. Through this program, we reached

			Underlying Community Issues	their homes, and many of their family problems came to light — things they wouldn’t normally share." (LHW Participant 3, Focus Group Discussion)
			Enhanced Counselling Skills through Psychoeducational Videos	When we conducted sessions, we would sometimes forget certain points. The videos explained everything in detail, step by step, which made it easier for us to communicate with participants. If we forgot something, the videos covered it in an organized manner. So, having the videos alongside was very helpful." (LHW Participant 5, Focus Group Discussion)<br><br>"We ourselves would watch the videos, and we would think, 'These are real issues, and this is how we should talk to people about resolving them.' The videos showed us how to educate people about these matters. It was very beneficial for us as well — watching the videos helped us understand how we could better develop the skills to support people in overcoming these challenges." (LHW Participant 9, Focus Group Discussion)

			Strong impact of Videos on Participants	What I liked the most was that, the videos in the application were very effective because the women would listen to them as if they were hearing from a doctor. It gave them a sense of reassurance and comfort." (LHW Participant 7, Focus Group Discussion) The videos provided significant guidance to the women. There were one or two who were completely illiterate, but they managed to follow along by looking at the visuals. When the videos played, they performed the exercises accordingly. We also explained some things to them, and they found it easier this way." (LHW Participant 4, Focus Group Discussion)

			Ease with WhatsApp support group	“the WhatsApp support group was very helpful. Specially when we had difficulty with session completion because of internet signals. If we couldn’t submit the session completion button our time was not recorded. We would simply take picture of session page and send it to WhatsApp group to supervisor. She could see the session number and patient name and upload our data at backend.” (LHW Participant 9, Focus Group Discussion)
		**LHW-Reported Unacceptable Aspects of TA-PM+**	Participant Discomfort with Personal Questions	"There was quite an issue when asking about someone's personal matters at home. For a woman, it was a big concern. In the beginning, many women felt uncomfortable, wondering why these questions were being asked." (LHW Participant 6, Focus Group Discussion)

				"Madam, the way these questions were asked, repeatedly, was difficult for them at first because they didn't understand it initially. Then sharing their problems and their solutions felt risky, as if their concerns might be revealed elsewhere. But when we assured them that we would maintain confidentiality, they gradually started opening up to us, and after that, it wasn't much of a problem." (LHW Participant 11, Focus Group Discussion)
			LHW Discomfort Conducting Sessions During or After Participants' Domestic Conflicts	"The environment in their homes was such that we would sometimes feel uncomfortable ourselves. If they were going through a personal issue, it didn't feel right to visit at that moment. Sometimes there would be arguments with their husbands or conflicts with their mothers-in-law. So, when we visited, we felt that they themselves were not relaxed at the time, and they wouldn't be able to respond properly to our questions." (LHW Participant 8, Focus Group Discussion)
			Difficult to engage Patients with high stress / asked for tangible benefits	"Many women complained that we were only making them do exercises. They said, 'You're not doing anything else with us — give us some medicine or something.'" (LHW Participant 4, Focus Group Discussion)<br><br>"If someone had a serious concern, they would say, 'You're just trying to convince us with words. What's the benefit of that? Give us some

				medicine, guide us properly, or connect us with a doctor.'" (LHW Participant 7, Focus Group Discussion)<br><br>"Madam, even when you visited, they said, 'These breathing exercises aren't helping us. Please help us — we are poor and need real support.'" (LHW Participant 3, Focus Group Discussion)
		**Participant reported unacceptable aspects of TA-PM+**	Watching video cannot solve our issues	"To be honest, we thought to ourselves, 'Are we just wasting our time? How will this help us? My problems can't be solved just by looking at a mobile phone...'" (Participant 3)
			Losing interest or motivation due to stress	"I tried it three times. But whenever there was a fight at home, I would forget everything... What's the point? When my husband scolds me, naturally, I forget everything — what else can I do, I alone can do nothing if my home environment doesn't change?" (Participant 5)
			Lack of follow up	"I didn't pay much attention because several days had passed. I thought, 'Who's going to come? No one comes to ask anyway. I didn't really understand that this was something beneficial for me, to be honest." (Participant 3)
**Suggestion to improve TA-PM+**	*This includes recommendations provided by participants and LHWs for improving the TA-PM+ intervention, addressing aspects such as content, delivery methods, technical issues, and suggestions for enhancing overall acceptability and effectiveness.*	**Suggestion to improve Digital app of TA-PM+**	Provision of Videos to Participants' Personal Devices	*"Like how videos come to us on WhatsApp, for example, then we can watch them at any time. While watching the videos during session, my little kid was attracted and kept coming*

				to me, so I couldn't concentrate." (Participant 15)
			Provision of PM+ Materials in Written Format/ Messages	"It's better if you write all these things down... Give each patient a page with the information written on it so they can read and understand it or send it as a message." (Participant 3)
			Translation of Complex Terms in Videos	"I was saying that some English words are used in it. They use such words that not everyone can understand. So my suggestion is that they should explain the meaning alongside, like if they use a word, they should also translate it and explain what it means." (Participant 15)<br>"...It would be better if there's a translation... Yes, this is just a suggestion — since we are less educated people, it would be better if there were no English words in between." (Participant 2)
			Offline functionality	"Make it easier — fix the internet issue... There were too many internet problems." (LHW Participant 4, Focus Group Discussion)<br>"Give us something that works without the internet so that if they want to implement this in other areas, they won't need internet access. It should work the same way for everyone." (LHW Participant 7, Focus Group Discussion)
			Concise and Precise Question Formulation	"The questions should be brief so that they take less time." (LHW Participant 6, Focus Group Discussion)

		**Suggestion to improve TA-PM+ delivery by participants**	Advocacy for Enhanced Participant Engagement	"My view is that you should first develop interest instead of directly starting a session or showing a video." (Participant 12)
			Need for Tangible or Monetary Incentives	"If all this is to be done, then first do something about our financial situation... When people's financial conditions improve, their mental peace will also improve. Everything will get better." (Participant 8)
			Access to Medical Check-Up for mental health in Facility	"Have them undergo a proper check-up to find out the cause of their anxiety. It could be that their blood pressure is high, which is putting strain on their mind, or they may be feeling stressed for other reasons." (Participant 11)
			Hybrid Session Format as Beneficial (Group and Individual)	"Call us in a group (weekly) and ask about our problems so that others can also understand these issues... But also talk to us separately because some problems are such that a person can't share them in front of everyone, while others can be shared openly." (Participant 15)
			Extension of Sessions to Include Spouses	"I say that someone should come — a madam who can also explain things to the men. That would be good." (Participant 5)<br>"I believe the videos should include content that encourages husbands not to get angry over small things. They should be taught to speak calmly with their wives. If a woman stays healthy, the children will be happy, and the husband himself will also be happy." (Participant 6)

			Splitting sessions to Control Session Duration	"That's exactly what I'm saying — the day you plan to show a video, only show the video. The day there's mobile work, focus solely on that. And when you're taking a session, just conduct the session that day. This way, the mind stays fresh. When the session drags on for too long, people start to get irritated." (Participant 2)
			Optimal Session duration-Half to an hour long	There should be a little more time for explanation… not too long, but at least 30 minutes. (Participant 14)<br><br>I believe the session should not exceed 30 minutes… Half an hour is enough for a person. At least, one can manage to take out 30 minutes. (Participant 3)<br><br>A person can spare at least 30-45 minutes a day for themselves… But think the time should be slightly increased. (Participant 15)
		**Suggestion to Improve TA-PM+ delivery by LHWs**	Referral Mechanism for Mental Health Issues	"They had small issues... so if we refer them somewhere, there should be a proper response — something positive that shows we actually sent them to the right place." (LHW Participant 5, Focus Group Discussion)
			Patient Recruitment by LHWs Based on Local Knowledge	"Madam, I suggest that next time when you select someone for a session, ask the workers themselves... They should personally identify patients who actually have some issues." (LHW Participant 10, Focus Group Discussion)

				"Madam, the workers are from the field, and they know better who among the women really needs this and who doesn't." (LHW Participant 10, Focus Group Discussion)
			Preference for Health House for conducting sessions	There was no internet issue at the Health House because WiFi was available, and there was no noise or disturbance. When we conducted sessions at participants' homes, interruptions were frequent—guests would arrive, or children would come in, causing them to get up repeatedly. (LHW Participant 4, Focus Group Discussion)<br><br>When I went to a client's home, there was a significant internet issue, so I had to cancel the session. Later, she came to my home in the evening, and we conducted the session there. I mentioned that internet issues cause time wastage for both participants and us, which hinders the success of our objective. (LHW Participant 6, Focus Group Discussion)
