# Supplementary material for: Feasibility and acceptability of technology-assisted problem management plus (TA-PM+) in community settings in Pakistan: a pre-post mixed-methods study
Source: Sci Rep. 2026 Apr 30;16:14153. doi: 10.1038/s41598-026-49596-8 (PMC13139419; doi:10.1038/s41598-026-49596-8)
Supplement: Supplementary file 5 — Supplementary Material 5 [file 41598_2026_49596_MOESM5_ESM.pdf]

Covariate Analysis of GAD-7 and PHQ-9 scores.

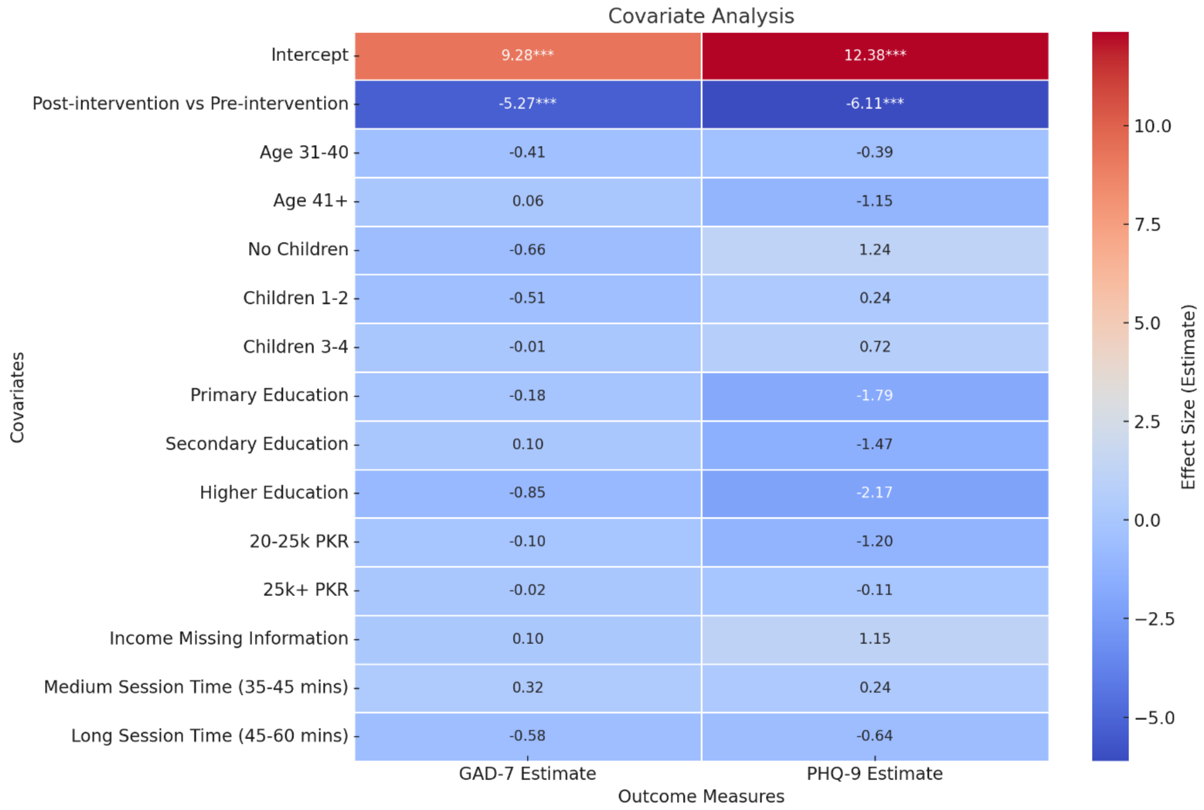

GAD-7: Generalized Anxiety Disorder-7,
PHQ-9: Patient Health Questionnaire-9,
****: p < 0.001 and denote significance,*
PKR: Pakistani Rupee.

Sensitivity Analysis (without Participants of LHW 8 and 9)

Measure	N	Baseline Mean (SD)	Follow-up Mean (SD)	Mean Difference	Difference SD	t(df)	p -Value	Signed Rank Statistic (S)	p (Wilcoxon)
PHQ-9	56	12.73 (5.42)	5.61 (4.72)	7.12	7.73	6.64 (55)	< .001	177.5	< .001
GAD-7	56	8.82 (4.20)	3.34 (3.41)	5.48	5.99	6.64 (55)	< .001	145	< .001
