# Supplementary material for: Feasibility and acceptability of technology-assisted problem management plus (TA-PM+) in community settings in Pakistan: a pre-post mixed-methods study
Source: Sci Rep. 2026 Apr 30;16:14153. doi: 10.1038/s41598-026-49596-8 (PMC13139419; doi:10.1038/s41598-026-49596-8)
Supplement: Supplementary file 6 — Supplementary Material 6 [file 41598_2026_49596_MOESM6_ESM.pdf]

Supplementary file 5: Mixed-method Triangulation of Quantitative and Qualitative Findings Mixed-method Triangulation of Quantitative and Qualitative Findings

Evaluation	Quantitative findings	Qualitative findings
Overall effectiveness		
	Overall mean GAD-7 reduction being 5.25 (SD: 1.8, $p<0.0001$) and PHQ-9 reduction being 5.75 (SD: 2.0, $p<0.0001$). The GAD-7 scores exhibited more consistent improvements across participants, reflecting the intervention's targeted focus on anxiety reduction strategies, particularly breathing exercises.	The most practiced strategy as highlighted by LHW and patient was breathing exercises "Whenever I feel like it, I definitely do it." (Participant 10)<br><br>"Yes, I am doing this (breathing exercise), that's why I remember it; those who are not doing it won't remember." (Participant 12)<br><br>"Now, whenever the lady (health worker) leaves after visiting our home, we lie down, sit on the bed, and do these exercises to relax." (Participant 4)

		"They were explaining about tension, that you should hold your breath for some time, hold it for a minute or so, then take deep breaths, and you will feel relief." (Participant 14)<br><br>"Since I started doing the exercises, especially the breathing one, I've noticed that my mind and depression have started to ease." (Participant 8)<br><br>"Yes, these breathing exercises. As I told you, I feel satisfied when I do these exercises." (Participant 15)<br><br>"They were taught breathing exercises, and it has brought significant improvement. They say they used to feel tension and fatigue, but now they

			don’t feel it anymore. They are fine now." (FGD LHW)
Internet Connectivity			
	Mental Health Outcomes (Mean)	Implementation Outcome	Qualitative findings
LHW 8	Gad 7 (5), PHQ-9 (5.5)	Session data time (missing for available)	Internet connectivity was identified as a major barrier, particularly for recording session time durations, which were essential for evaluating intervention fidelity. While all components of the app, including videos, functioned offline, sessions were marked as "not delivered" if the app failed to connect to the internet for data submission. Although this fidelity check provided valuable insights for evaluation, it posed a significant challenge for the LHWs, often disrupting their
LHW 9	GAD 7 (5.5) PHQ9 (6.5)	Session data Time (missing for all 6 participants)	

			workflow and adding to their stress.<br>“I don't know if it was an internet issue or something else, but our sessions were not getting submitted." (FGD, pg 19)<br>"Our sessions wouldn’t submit; sometimes we had to try 2-3 times to submit a single session. There were many such problems." (FGD, pg 19)<br>"When I went to their homes, the issue was weak signals, and when I submitted the session, it appeared successful, but when they checked the record, it was missing." (FGD, pg 20)<br>"The biggest issue was that the application didn’t work because there was no network inside people’s homes." (FGD, pg 11)

			"Due to signal issues, sessions didn’t submit, and when we tried again, the timer wouldn’t start, so the recorded session time was shorter." (FGD, pg 3) "When I visited their homes, there was a network issue, so I canceled the session and had them come to my home in the evening to complete it. I said that network issues waste their time and mine, which compromises the purpose of our sessions." (FGD, pg 28) "There were one or two instances where their neighbors had internet, so I connected to their Wi-Fi. Otherwise, mobile data didn’t work at all." (FGD, pg 36)
